# Eosinophils and eosinophilic immune dysfunction in health and disease

**DOI:** 10.1183/16000617.0150-2021

**Published:** 2022-01-26

**Authors:** David J. Jackson, Praveen Akuthota, Florence Roufosse

**Affiliations:** 1 Guy's Severe Asthma Centre, Guy's & St Thomas’ NHS Trust, London, UK; 2 School of Immunology & Microbial Sciences, King's College London, London, UK; 3 Division of Pulmonary, Critical Care, and Sleep Medicine, Dept of Medicine, University of California, San Diego, La Jolla, CA, USA; 4 Médecine Interne, Hôpital Erasme, Université Libre de Bruxelles, Brussels, Belgium

## Abstract

The functions ascribed to eosinophils have classically been limited to host defence against certain parasitic infections and potentially deleterious effects in the setting of specific diseases that are associated with elevated eosinophil counts in blood and/or tissue. The ability to induce eosinophil depletion either experimentally in animal models or through targeted therapies in humans has extended our understanding of the roles played by eosinophils in health and homeostasis as well as in disease pathogenesis. When associated with human disease aetiology, the eosinophil takes on a pathogenic rather than a protective role. This maladaptive response, called “eosinophilic immune dysfunction” herein, appears central to exacerbation pathogenesis and disease control in severe asthma and may be involved in the aetiology of other eosinophil-related conditions ranging from organ-system-limited diseases such as phenotypic subsets of chronic obstructive pulmonary disease and chronic rhinosinusitis with nasal polyposis to more broadly systemic diseases such as eosinophilic granulomatosis with polyangiitis and hypereosinophilic syndrome. In this review, we describe the evidence supporting eosinophilic functions related to health and homeostasis and explore the contribution of eosinophilic immune dysfunction to human disease.

## Abstract 2

**The current era of therapies that specifically target eosinophils has yielded new insights into the functional significance of eosinophils in the maintenance of health and the effects of eosinophilic immune dysfunction on disease pathogenesis.** https://bit.ly/3pVPLR5

## Introduction

In conjunction with the European Respiratory Society International Congress in 2020, an expert faculty participated in a sponsored symposium that described emerging evidence for the functional role of eosinophils in health maintenance and eosinophilic immune dysfunction as a contributor to disease pathogenesis across an array of inflammatory diseases. In this review, the presenters from the symposium revisit their original content and provide additional insights derived from the subsequent publication of relevant clinical trial data and analyses.

This is a fascinating time for scientists and clinicians interested in diseases that involve eosinophilic immune dysfunction, including severe asthma, chronic rhinosinusitis with nasal polyposis (CRSwNP), gastrointestinal diseases such as eosinophilic esophagitis (EoE), and systemic diseases such as eosinophilic granulomatosis with polyangiitis (EGPA) [1–4]. Our understanding of these diseases and the contribution of eosinophils to disease pathogenesis has expanded rapidly over recent years, thanks in part to the availability of therapies that selectively and safely target eosinophils [5]. The creation of human eosinophil “knockouts” using biologic therapies has offered a unique opportunity to elucidate the role of the eosinophil across a variety of disease states [5, 6]. Moreover, demonstration of clinical efficacy has led to the approval of such therapies for an expanding list of specific conditions, frequently allowing for dose reduction of oral corticosteroids (OCS) [7–13], a drug class that is often implemented for disease management yet is associated with significant morbidity due to its association with hypertension, type 2 diabetes, gastric ulcers, obesity, osteoporosis, fractures, cataracts, glaucoma and sarcopenia, among other unfavourable effects [14–16]. In the following sections, we will review the role of eosinophils in human health and disease at a general level and describe the evidence supporting eosinophilic immune dysfunction as a deleterious factor in chronic airway inflammation and in systemic inflammatory diseases. Where relevant, data from clinical trials of currently licensed therapies that directly target the eosinophil (*e.g.*, benralizumab, mepolizumab, and reslizumab) will be presented.

## The role of eosinophils in health and disease development

Eosinophils are cells of the myeloid lineage that develop in the bone marrow in response to a specific combination of transcription factors and cytokines [17]. Upon differentiation and maturation, eosinophils leave the bone marrow, enter the circulation, and are rapidly recruited to peripheral tissues. Less than 1% of the total pool of eosinophils in the body are found in the circulation, where they represent <5% of white blood cells. The main tissues in which eosinophils are recruited under homeostatic conditions are the digestive tract, adipose tissue, lung, mammary gland, thymus, and uterus; the vast majority of eosinophils are found in the mucosal lining of the digestive tract.

Eosinophils are granulocytes, and as such contain numerous granules of varying composition [18]. Cationic granule proteins mediate many of the cytotoxic effects of eosinophils and are believed to play a role in host defence and to contribute to inflammation. Major basic proteins and eosinophil cationic proteins display direct cytotoxic effects on various cell types including endothelial and epithelial cells, while eosinophil peroxidase (EPO) generates reactive oxygen species that contribute to cytotoxicity. Eosinophil granules also contain cytokines, chemokines, growth factors, and lipid mediators that are selectively released in response to different stimuli.

### Eosinophils and inflammation

**FIGURE 1:**
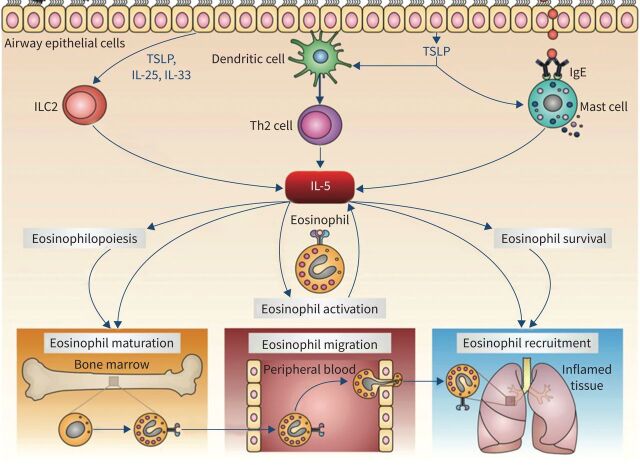
The role of interleukin (IL)-5 in eosinophil biology. T helper type 2 (Th2) lymphocytes, group 2 innate lymphoid cells (ILC2), and mast cells release IL-5 in response to a variety of triggers, including inhaled allergens, respiratory viruses, and airborne pollutants. IL-5 promotes eosinophil maturation, migration out of the bone marrow, and recruitment to peripheral sites. IgE: immunoglobulin E; TSLP: thymic stromal lymphopoietin. Reproduced from Pelaia *et al.* [21].

Eosinophilic inflammation can be driven by T-cells, more specifically T helper type 2 (Th2) cells, which differentiate in response to dendritic cell presentation of certain antigens such as those derived from helminths or allergens [19]. Th2 cells produce several cytokines, including interleukin (IL)-5, a key player in the proliferation and maturation of eosinophil progenitors expressing the high-affinity IL-5 receptor (IL-5R) alpha chain in addition to the common beta chain (figure 1) [20, 21]. Binding of IL-5 to its receptor activates a network of intracellular signalling pathways, including the Janus kinase–signal transducer and activator of transcription pathway, which is involved in the transcriptional regulation of genes that control eosinophil proliferation. In addition to Th2 cells, other cells such as group 2 innate lymphoid cells can function as an early source of IL-5 in certain diseases. Mature eosinophils also express IL-5R and are the only cell type that expresses the IL-5R alpha chain at high levels in humans. IL-5 acts on mature eosinophils in a number of ways, including promoting egress from the bone marrow into the circulation, synergising with chemotactic factors to attract eosinophils to peripheral tissues, prolonging eosinophil survival in tissue, and inducing eosinophil activation and degranulation.

Eosinophils respond to a number of mediators in addition to IL-5 (*e.g*., cytokines, chemokines, lipid mediators) through the expression of a vast array of specific receptors [22]. Eosinophils have pattern recognition receptors for pathogen-associated molecular patterns and damage-associated molecular patterns, and can bind immunoglobulin chains, including IgA and IgE. In recent years, the alarmin IL-33 has emerged as an important mediator of eosinophilic inflammation in atopic disease and chronic obstructive pulmonary disease (COPD) [23, 24]. Although both IL-5 and IL-33 are involved in type 2 immune responses, preliminary evidence suggests that eosinophil functions differ when they are activated by IL-5 *versus* IL-33 [23]. These types of observations suggest the existence of plasticity in eosinophil development and activation that may result in eosinophils with distinct characteristics. Further study is needed to establish the presence and potential significance of eosinophil endotypes.

### Physiologic roles of eosinophils

Classically, eosinophils are known to combat helminthic infections and to induce tissue damage in the context of allergy [25, 26]. During migration of helminthic larvae in tissues, an antigen-specific type 2 immune response develops, resulting in eosinophil expansion and IgE-type immunoglobulin production. The IgE antibodies bind to their target antigen and activate eosinophils following engagement of eosinophil-membrane-expressed fragment crystallisation receptor epsilon, resulting in the release of cytotoxic substances including granule proteins in close proximity to the helminth, which damage the cell membrane [25]. The same cytotoxic substances, when released by eosinophils in tissue, damage nearby cells [26]. For example, it has been shown in severe asthma that numerous activated eosinophils are observed in close proximity to bronchial epithelium with marked structural damage.

Several homeostatic functions have been described for eosinophils, the vast majority of which have been demonstrated in murine models [22]. The mouse models used for most of these studies are genetically devoid of eosinophils, including the PHIL transgenic line, in which expression of a cytocidal protein is under the control of the EPO gene promoter, thus eliminating cells that would normally express EPO (*i.e*., eosinophils) [27]. Another mouse model, ΔdblGATA, has a deletion of a high-affinity GATA-binding site from the promoter region of the GATA-1 gene, thereby disrupting expression of GATA-1, which plays a key role in eosinophil development [28]. Using these models, it has become apparent that mice lacking eosinophils appear to have a normal lifespan and develop no specific illnesses or abnormalities. In addition, even the dogma that eosinophils play a central role in defence against helminth infections has been drawn into question, with studies showing either no difference in outcome following infection compared with wild-type controls or an improved outcome in eosinopenic mice, suggesting that certain parasites may, in fact, rely on eosinophils for their survival [29, 30].

Other studies suggest that eosinophils play a role in host defence against viral and bacterial pathogens and fungi [22]. Indeed, a decrease in peripheral eosinophil counts has long been noted in association with bacterial infections, a finding that is attributed to accumulation of eosinophils at the site of inflammation coupled with suppression of eosinophil migration from the bone marrow [31]. However, the results of some experimental infection models are difficult to align with observations of controlled studies of eosinophil-depleting anti-IL-5/5R biologics in humans, which do not appear to show increased rates of viral, bacterial, or helminth infections [5, 6]. Moreover, although eosinopenia has been frequently observed in hospitalised patients with coronavirus disease 2019 (COVID-19) and has been linked to patient outcomes [32], emerging data indicate no increase in hospital admissions for COVID-19 amongst patients with asthma receiving eosinophil-depleting biologic therapies [33–37]. In fact, a potential beneficial effect in terms of a milder course of disease has been postulated for some patients. In addition, the occurrence of exacerbations in patients with asthma, a process that is commonly induced by viral infection, is significantly reduced by eosinophil-depleting biologic therapies [10, 38–41]. Together, these data do not indicate that the absence of eosinophils impairs host defence against helminths, viruses, bacteria, or fungi in humans.

Eosinophils have also been implicated in immune system homeostasis, acting at different levels of B-cell, plasma cell, and T-cell functions, as well as in the regulation of gut microbiota [22, 42]. Although a functional role of eosinophils in homeostasis in humans remains unclear, their role in human disease has become better characterised from observations in several conditions, particularly hypereosinophilic syndrome (HES), wherein eosinophils are central to disease pathogenesis [43, 44]. HES is a rare disease in which blood and tissue eosinophil counts are elevated; main target organs include the lungs, skin, digestive tract, and heart. Eosinophils are directly involved in structural and dysfunctional alterations that are associated with a wide array of clinical manifestations in HES (table 1) [43, 44]. Typically, and by definition, the damage is directly related to the presence of excess activated eosinophils in affected tissue.

**TABLE 1:** Wide-ranging clinical manifestations of hypereosinophilic syndromes [43, 44]

**General** Fatigue, myalgia, weight loss, fever	**Ocular** Retinal micro-emboli, choroidal inflammation
**Pulmonary** Asthma, lung infiltrates, fibrosis	**Splenic** Splenomegaly
**Gastrointestinal** (Gastro-)enteritis, colitis	**Sino-nasal cavities** Chronic rhinosinusitis, polyposis
**Hepatic** Hepatitis, cholangitis	**Soft tissue/rheumatologic** Angioedema, fasciitis, myositis, synovitis, arthritis
**Neurologic** Embolic stroke, encephalitis, peripheral neuropathy	**Dermatologic** Pruritis, eczema, dermatitis, urticaria, erythroderma
**Vascular** Arterial/venous thrombosis, microvascular damage, vasculitis	**Cardiac** Myocarditis, intracavitary thrombus, subendocardial fibrosis, valve entrapment, pericarditis

Beyond HES, there is an increasingly large group of disorders in which elevated eosinophil counts are present in blood and/or tissue and are believed to contribute, at least in part, to clinical manifestations and tissue damage (table 2) [6, 22, 44–46]. Targeting eosinophils as a means to limit damage is an active area of investigation, as will be described in subsequent sections.

**TABLE 2:** Eosinophilic immune dysfunction is implicated in a spectrum of disorders^#^ [6, 22, 44–46]

**Category**	**Diseases**
**Lung**	Severe eosinophilic asthma, EGPA, COPD, allergic bronchopulmonary aspergillosis, chronic eosinophilic pneumonia
**Skin**	Atopic dermatitis, contact dermatitis, chronic spontaneous urticaria, bullous pemphigoid, eosinophilic cellulitis, eosinophilic fasciitis, Kimura disease, severe drug reactions (DRESS: drug reaction with eosinophilia and systemic symptoms)
**Gastrointestinal**	Eosinophilic esophagitis, eosinophilic gastritis, eosinophilic gastroenteritis, eosinophilic colitis, inflammatory bowel disease, radiation-induced enteropathy
**Upper airways**	Chronic rhinosinusitis with or without nasal polyposis
**Rheumatologic**	Rheumatoid arthritis, vasculitis, juvenile temporal arteritis
**Neuromuscular**	Neuromyelitis optica, Duchenne muscular dystrophy
**Neoplastic/paraneoplastic**	Chronic eosinophilic leukaemia, mastocytosis, T-cell lymphoma, solid tumours (adenocarcinoma)
**Primary immunodeficiencies**	Omenn syndrome, autosomal dominant hyper-IgE syndrome (formerly Job syndrome), CARD9 mutations

CARD9: caspase recruitment domain containing protein 9; COPD: chronic obstructive pulmonary disease; EGPA: eosinophilic granulomatosis with polyangiitis; IgE: immunoglobulin E. ^#^: this list includes examples of diseases for which eosinophilic dysfunction is potentially involved and is not comprehensive.

### Eosinophils in health and disease

Our perspective on how eosinophils contribute to health and disease has progressed enormously over the past 20 years; however, much of the data in relation to health is conflicting. Murine models suggest a role for eosinophils that extends far beyond their alleged function in defence against parasitic infections: they may be involved in host defence against other types of organisms and appear to contribute to gut homeostasis as well as maintenance of an intact immune system [17, 22]. In disease states, hypereosinophilic conditions can be subdivided according to whether eosinophils are the major subtype involved in organ damage, such as HES or chronic eosinophilic pneumonia, or contribute at least partially to disease pathogenesis as part of a complex network of interactions between cells and mediators, as is observed in asthma and other diseases with an allergic or autoimmune component.

Given the roles that eosinophils appear to play in health in murine models, should there be concern for therapies targeting eosinophils in humans? We do not have the answer to that question yet, but there are reassuring indirect data. Overall, global health does not seem to be affected in mice who are completely devoid of eosinophils or in humans lacking eosinophils [6, 47]. Data on the effects of eosinopenia in humans comes primarily from monoclonal antibodies developed for the treatment of eosinophil-mediated diseases, three of which have received marketing approval: mepolizumab, reslizumab, and benralizumab. Mepolizumab and reslizumab, both IL-5-targeted therapies, reduce (but do not fully deplete) the number of eosinophils, whereas benralizumab, an IL-5R-targeted therapy, almost completely depletes eosinophils [6]. Even with the near-complete absence of eosinophils, no safety concerns regarding malignancy, opportunistic infections, or helminth infections have arisen in the more than 8000 patients who received benralizumab as part of the clinical development programme or in the numerous patients treated post-approval [6]. The ability to maintain normal functionality in the absence of eosinophils is not well understood but may involve redundancy in host defence mechanisms (*e.g*., mast cells fulfilling the role of defence against parasitic infections), sparing of certain eosinophil types such as regulatory or homeostatic eosinophils by IL-5/IL-5R-targeted treatments, and/or possible persistence of IL-5R-expressing eosinophils in tissue.

Although our understanding of eosinophil function in healthy individuals and those with hypereosinophilic conditions has increased, many questions remain unanswered. With the recent widespread availability of eosinophil-targeted therapies, we will likely sharpen our understanding of the role of eosinophils in health and homeostasis in humans (*e.g*., the ability to respond to vaccines, to ensure tumour surveillance and to carry out host defence against various pathogens), determine whether eosinophil subsets (resident *versus* inflammatory) that are found in mice also exist in humans, and clarify the relative contribution of eosinophils to complex, multi-mediator, multi-pathway inflammatory diseases.

## Eosinophils as the effector cell in chronic airway inflammation

### Linking eosinophils and asthma

Among diseases associated with eosinophilic immune dysfunction, asthma has been a rich subject of study from both therapeutic and research perspectives. We have known for a long time that the eosinophil appears central in the immune dysfunction of asthma. In 1958, research from Harry Marrow Brown suggested that unless patients had eosinophils in their sputum, prednisolone was not effective [48]. In 1975, Horn *et al.* [49] demonstrated that the “total eosinophil count reflects asthmatic activity and is useful for regulating steroid dosage and for early detection of exacerbations”. In 1990, Bousquet *et al.* [26] described the relationship between eosinophil counts (in blood and sputum) and asthma severity and presented histologic evidence of eosinophils in close proximity to destructured airway epithelium, suggesting a direct cytotoxic role for eosinophils in asthma.

The link between eosinophils and asthma re-emerged in 2002 with the publication of a study by Green *et al*. [50], which made it clear that a therapeutic approach targeting eosinophilic inflammation resulted in better asthma outcomes. In this study, patients with moderate to severe asthma were randomised to either management in accordance with standard asthma guidelines or aiming for normalisation of sputum eosinophil count. The decision to escalate or de-escalate therapy in the latter group was based purely on the objective measure of inflammation as indicated by sputum eosinophil count. Patients for whom the decision to alter therapy was based on eosinophilic inflammation experienced fewer severe asthma exacerbations compared with standard management.

**FIGURE 2:**
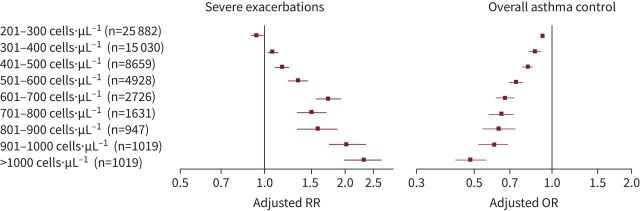
Relationship between eosinophil counts and severe asthma exacerbations or overall asthma control. Data are adjusted rate ratios (RRs) with 95% confidence intervals for severe exacerbations and adjusted odds ratios (ORs) with 95% confidence intervals for overall asthma control for patients assigned to eosinophil count categories compared with the reference category of eosinophils ≤200 cells·µL^−1^. Analyses were adjusted for age, sex, body mass index, smoking status, and Charlson comorbidity index. Adapted from Price *et al.* [51].

In 2015, Price *et al.* [51] published data from an historical cohort of more than 130 000 patients with asthma treated in the primary care setting in the UK. This study showed that blood eosinophil count is both a biomarker of severe asthma exacerbation risk and of asthma control. As blood eosinophil counts increased, the risk of severe exacerbations increased and the ability to maintain good asthma control decreased (figure 2).

### Eosinophilic inflammation in asthma

A 2011 study by Wang *et al.* [52] demonstrated that even patients who appear non-eosinophilic in their stable state had evidence of eosinophilic inflammation in sputum when examined during an asthma exacerbation. 86% of patients were either purely eosinophilic or had mixed eosinophilic/neutrophilic sputum during an exacerbation. This contrasts with the 22% of patients who appeared eosinophilic in the stable state. These observations have ramifications for clinical practice because phenotype is often based on a single blood eosinophil measurement taken when the patient is well. It is crucial to see an exacerbation-prone patient during an exacerbation event to determine whether the patient actually has active eosinophilic disease.

The presence of eosinophils in the airways of patients with asthma may also be affected by concurrent viral infection. A 2014 study assessed the influence of rhinovirus, probably the most common trigger for asthma exacerbations, on airway eosinophilia [53]. 28 patients with asthma and 11 healthy controls were inoculated with rhinovirus-16. Patients with asthma experienced induction of type 2 (T2) inflammation, as illustrated by increased nasal IL-5 concentrations and elevated eosinophil counts in bronchoalveolar lavage fluid measured 4 days post-inoculation. This was not observed in healthy controls and highlights the difference between the immune response to rhinovirus infection in patients with *versus* without asthma.

We also know that eosinophilic inflammation appears to be very important in the context of mucus plugging in airway disease. Dunican *et al.* [54] demonstrated that: 1) mucus plugging was common, 2) it persists for many years, and 3) it is associated with significant T2 inflammation. Patients with the highest degree of mucus plugging on computed tomography had the highest levels of airway eosinophilia. Recent data implicate Charcot–Leyden crystals within mucus as a T2 adjuvant that promotes inflammation [55]. Dunican *et al.* [54] also found that a high mucus plug score occurred more frequently in patients with reduced *versus* normal lung function. It is interesting to note that a significant improvement in lung function occurred in patients with severe asthma who received benralizumab in phase 3 studies [38, 39, 56]. Improvements were observed as early as week 2 and were maintained throughout the studies. Further research is needed to understand whether this may reflect a reduction in eosinophil-rich mucus plugging in patients with severe eosinophilic asthma (SEA).

### Therapies targeting eosinophils in severe asthma

To really understand how important eosinophilia is in the context of severe asthma, data from real-world studies and clinical trials of drugs such as mepolizumab and benralizumab are invaluable. In two phase 3 studies, mepolizumab decreased the asthma exacerbation rate (AER) by 32% to 53% compared with placebo in patients with SEA [10, 40]. In a 1-year, open-label extension study, the mean AER was 0.90 (95% CI 0.78–1.04) in patients who received mepolizumab in the original and extension studies, and 54% were completely exacerbation-free in year 2 [57]. In the equivalent, open-label extension study for benralizumab, which included data from 1926 patients with severe, uncontrolled asthma, the AER in patients who had received benralizumab 30 mg every 8 weeks (Q8W) in both the original and extension studies was 0.46 (95% CI 0.39–0.53) [58]. In addition, 74% of patients who received benralizumab 30 mg Q8W in both the original and extension studies were exacerbation-free in year 2 [58], highlighting the key role of eosinophils in exacerbation pathogenesis.

#### Treatment response and atopic status

**FIGURE 3:**
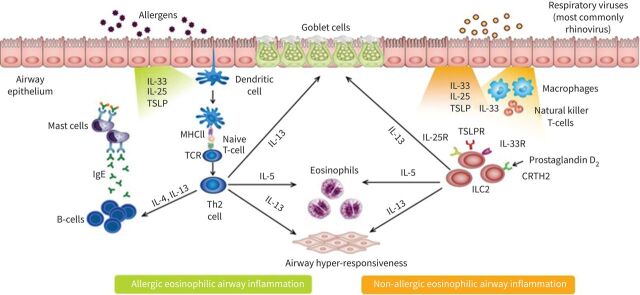
Pathways driving eosinophilic immune dysfunction in asthma. The illustration highlights the common elements and differences in signalling cascades that result in eosinophilic inflammation caused by allergic *versus* non-allergic sources. Biologic therapies developed for the treatment of asthma, including anti-eosinophil (interleukin (IL)-5/5R) therapies, anti-immunoglobulin E (IgE) therapies, and anti-IL-4R therapies, target different points in these pathways. CRTH2: prostaglandin D2 receptor 2; ILC: innate lymphoid cell; MHC: major histocompatibility complex; R: receptor; TCR: T-cell antigen receptor: Th2: T helper type 2; TSLP(R): thymic stromal lymphopoietin (receptor). Adapted from Brusselle *et al.* [59].

Broadly speaking, there are two main pathways that are believed to promote eosinophilic inflammation in asthma: an adaptive allergic pathway and an innate non-allergic pathway (figure 3) [59]. These pathways can be active simultaneously. A large real-world study conducted in Japan evaluated treatment with omalizumab, an anti-IgE monoclonal antibody, in 2723 patients with severe allergic asthma [60]. Despite omalizumab and the appropriate clinical phenotype of severe allergic asthma, 42% of patients continued to have ≥2 exacerbations per year and more than one-quarter of patients had ≥4 exacerbations per year. This suggests that targeting IgE rather than eosinophilia may be insufficient to prevent exacerbations for a large proportion of patients.

Data indicate that response to anti-IL-5/5R monoclonal antibodies is independent of atopic status. A *post hoc* analysis of a phase 2b/3, placebo-controlled mepolizumab study performed by Ortega *et al.* [61] parsed patients in two ways: into quartiles by baseline IgE and by atopic status. Patients in the lowest quartile of IgE (≤70 kU·L^−1^) experienced the same reduction in exacerbation rate with mepolizumab *versus* placebo as patients in the highest IgE quartile (≥430 kU·L^−1^). Similar results have been reported for benralizumab. A *post hoc* analysis was conducted to assess the relationship between baseline IgE and exacerbation rates in pooled data from phase 3 studies of benralizumab [62]. Two main observations related to IgE came from this analysis. First, IgE itself is not a biomarker of risk. In the placebo group, the exacerbation rate was constant regardless of the baseline IgE concentration. Second, in patients who received benralizumab, the AER was lower than placebo and <1 across the range of baseline IgE concentrations. In contrast, as the baseline eosinophil count increased, the risk of exacerbations increased in the placebo group and the effect size (the difference between exacerbation rates in the benralizumab and placebo groups) became larger.

#### Steroid-sparing studies

Other illustrative findings regarding the importance of eosinophilic inflammation in asthma come from phase 3 steroid-sparing studies. Among adults with severe asthma randomised to treatment with benralizumab (30 mg every 4 weeks (Q4W) or Q8W) or placebo for 28 weeks, 52% of patients who received benralizumab Q8W were able to completely stop maintenance OCS use [7]. Despite this, the AER was 72% lower in patients receiving benralizumab Q8W *versus* placebo (0.54 *versus* 1.83; p<0.001). Real-world data support these findings, with a recent study demonstrating a 73% reduction in AER during benralizumab treatment compared with the year prior in patients with SEA [63]. Of patients receiving maintenance OCS at baseline, 51% were able to discontinue OCS during benralizumab treatment. A mepolizumab study also showed reductions in both OCS use and exacerbation rates but to a lesser extent compared with benralizumab data, with only 14% of patients able to completely discontinue OCS use and a 32% relative reduction in AER compared with placebo (1.44 *versus* 2.12; p=0.04) [10]. What does this tell us? Systemic steroids broadly dampen T2 inflammation, whereas a targeted drug such as benralizumab depletes eosinophils with only a minimal effect on other T2 pathways [64]. The fact that many of these patients were able to discontinue OCS completely and be exacerbation-free indicates that a major reason patients needed OCS was to suppress eosinophilic inflammation.

Steroid sparing may extend to inhaled corticosteroids (ICS) as well. Over the course of a 1-year study, patients with SEA who received mepolizumab and remained adherent to ICS experienced a reduction in exacerbations, whereas the exacerbation rate in patients with poor ICS adherence failed to improve [65]. In contrast, data from a similar study demonstrated that significant reductions in exacerbations with benralizumab were evident regardless of level of ICS adherence [66]. It is possible that this difference may reflect partial *versus* complete eosinophil depletion with these two therapies; however, further prospective studies are required to investigate this. A related ongoing, phase 4 study is evaluating whether benralizumab can minimize exposure to ICS and OCS while maintaining asthma control and reducing exacerbations in patients with SEA (ClinicalTrials.gov identifier: NCT04159519).

### Eosinophils and other airway diseases

Emerging data support a role for eosinophilic immune dysfunction in airway diseases other than asthma. COPD, for example, has not traditionally been considered an eosinophilic disease; however, there are some patients with COPD who have evidence of eosinophilic inflammation and, from an inflammatory point of view, are hard to distinguish from asthma [67]. Pooled analyses of data from studies of ICS added to long-acting β-agonists in patients with COPD have shown that blood eosinophil counts were predictive of exacerbations and response to ICS treatment [68, 69]. Although clinical trial results for eosinophil-depleting therapies in COPD have been mixed [70, 71], a correlation between higher baseline blood eosinophil counts and greater on-treatment reduction in exacerbations has been observed [72, 73], suggesting that eosinophils have at least a moderating effect on COPD pathogenesis for a subset of patients.

A role for eosinophilic inflammation is more established for CRSwNP based on histologic evidence of tissue eosinophilia, correlations between eosinophil counts and clinical outcomes, and evidence of therapeutic benefit from therapies that target eosinophils [56, 74–77]. In a phase 3 clinical trial, mepolizumab significantly improved nasal polyp score and reduced nasal obstruction compared with placebo in patients with CRSwNP [78]. Improvements in nasal symptoms (as measured by Sinonasal Outcome Test-22 (SNOT)22 score]) with mepolizumab had previously been reported for patients with SEA and comorbid nasal polyps [77]. Similarly, in a prespecified subgroup analysis from a phase 3b study, SNOT-22 score was significantly reduced in patients with SEA and comorbid nasal polyps who received benralizumab *versus* placebo [56]. Improvement was observed as early as week 4 and was maintained through the end of the study (week 24).

These data combined with the wealth of evidence in asthma affirm the importance of eosinophils as effector cells in airway disease, the extent of which has not been fully explored. Further clinical data in conditions such as COPD and CRSwNP will help identify endotypes that distinguish patients for whom eosinophils are significant contributors to disease pathogenesis and would, therefore, benefit from therapies targeting eosinophilic immune dysfunction.

## Eosinophils as the effector cell in systemic inflammatory diseases

Among systemic diseases in which eosinophilic immune dysfunction is a likely contributing factor, EGPA, previously known as Churg–Strauss syndrome, is one of the most well characterised. EGPA was first described by Jacob Churg and Lotte Strauss in the 1950s based on observations from autopsy and clinical histories of patients with a syndrome that included severe asthma and rhinosinusitis, hypereosinophilia, pneumonia (reflecting lung infiltrates), and vasculitis (characterised as eosinophilic arteritis and granulomas) [79]. Since the 1950s, there has been a greater understanding of other systemic manifestations of EGPA; it is not just a disease of the lungs, but a disease that involves multiple organ systems, including the sinuses (nasal polyposis, rhinitis with sinus and nasal inflammation), kidneys (glomerulonephritis, renal dysfunction, renal failure), gastrointestinal system, heart, nervous system (*e.g*., mononeuritis multiplex with foot drop and sensory changes), skin, and vasculature [80]. In the rheumatology literature, EGPA is considered a systemic vasculitis and is categorised as an anti-neutrophil cytoplasm antibody (ANCA)-associated small vessel vasculitis [81]. This classification highlighted that the spectrum of EGPA can include manifestations that are very much like other forms of vasculitis, with organ involvement of the gastrointestinal tract and kidneys. However, this classification does not acknowledge the presence of asthma and other airway components of EGPA or the frequent absence of ANCA in this disease. Whether EGPA is a systemic vasculitis, a variant of severe asthma, or both depends on whether it is viewed through a rheumatology or respiratory lens.

The diagnosis of EGPA has been a controversial topic over the years in terms of whether pathology with biopsy is required for definitive diagnosis or whether a clinical diagnosis based on the presence of syndromic components is sufficient [82]. Several groups have developed diagnostic criteria for EGPA, including the Chapel Hill, American College of Rheumatology (ACR) and Lanham criteria [81, 83, 84]; however, the phase 3 mepolizumab clinical trial diverged from these standards, defining EGPA by the presence of asthma and eosinophilia (>1000 cells·µL^−1^ and/or >10% of leukocytes) in addition to at least two of the following: positive biopsy findings (eosinophilic vasculitis, perivascular eosinophilic infiltration, eosinophil-rich granulomatous inflammation), neuropathy (mononeuritis multiplex or polyneuropathy), non-fixed pulmonary infiltrates, sinonasal abnormality (rhinosinusitis with or without nasal polyps), cardiomyopathy, glomerulonephritis, alveolar haemorrhage, palpable purpura, or a positive test for ANCA (myeloperoxidase or proteinase 3) [85]. A positive ANCA test is not mandatory and is present in only a subset (approximately 35–40%) of patients with EGPA [82]. The diagnosis of EGPA is made more challenging by its phenotypic overlap with other eosinophilic conditions such as HES, eosinophilic asthma, and eosinophilic pneumonia [82].

### Anti-IL-5 therapies in EGPA

Although EGPA is a hypereosinophilic disease, with elevated levels of IL-5 and eosinophils observed on biopsy of perivascular and vascular lesions [86], the relevance of these findings for overall disease pathogenesis was not previously known given the observed involvement of other immune pathways such as IL-17 and B-cells. A 2019 study provided insight into the potential pathogenic mechanism for eosinophils in EGPA. Mukherjee *et al.* [87] reported that immunoglobulins from ANCA-positive sputum of patients with EGPA (regardless of serum ANCA status) induced eosinophil cytolysis and the formation of extracellular DNA traps (*i.e.*, EETosis). A subsequent study confirmed the presence of EETosis in tissue from patients with EGPA [88].

Studies of anti-IL-5/IL-5R therapies further support eosinophilic immune dysfunction as a part of the pathobiology of EGPA. In a phase 3 clinical trial, patients who received mepolizumab were significantly more likely than placebo-treated patients to experience remission (odds ratio, 5.91 (95% CI 2.68–13.03); p<0.001) and less likely to have a relapse (hazard ratio, 0.32 (95% CI 0.21–0.50); p<0.001) [85]. Based on the strength of these data, mepolizumab was approved by the US Food and Drug Administration in 2017 for the treatment of adult patients with EGPA.

Benralizumab is also being studied in EGPA. In a small study conducted by Nanzer *et al*. [89], 11 patients meeting the 1990 ACR criteria for EGPA and who were receiving maintenance OCS initiated treatment with benralizumab Q8W [11]. During 24 weeks of treatment, median OCS dose was reduced by 50%. A 40-week, open-label pilot study similarly reported a decrease in maintenance OCS dose (46%) during benralizumab treatment in 10 patients with EGPA, half of whom were able to completely discontinue OCS [12]. Reslizumab, by a similarity in mechanism of action, is a plausible agent for EGPA as well. In an open-label pilot study of reslizumab 3 mg·kg^−1^ in 10 patients with EGPA, a decrease was observed in use of maintenance OCS [90].

Additional clinical data for these agents, including an ongoing phase 3 noninferiority study comparing benralizumab with mepolizumab in patients with EGPA (ClinicalTrials.gov identifier: NCT04157348) will provide further insights into the efficacy and safety of these agents for EGPA. It is still unclear, however, whether eosinophils contribute to the vasculitic inflammation/flares observed in EGPA and whether eosinophil-targeted therapy will impact this serious component of EGPA. Achievement of a Birmingham Vasculitis Activity Score of 0, indicating the absence of vasculitis, among a greater proportion of patients receiving eosinophil-depleting therapy compared with placebo in clinical trials points to a pathogenic role for eosinophils in vascular inflammation [85]. Real-world data on long-term responses to IL-5/IL-5R-targeted treatment in EGPA will be informative for understanding the contribution of eosinophils to vasculitis.

### Hypereosinophilic syndrome

HES, which is defined by the presence of absolute eosinophil counts >1500·µL^−1^ with evidence of eosinophil-associated organ damage and/or dysfunction, encompasses several variants, including myeloid HES, which is driven by proliferation of myeloid precursors in an abnormal fashion that may or may not be associated with a demonstrable/specific gene mutation; lymphoid HES, which often results from T-cell clones that overproduce eosinophil-inducing factors such as IL-5 or other cytokines (*e.g*., IL-3, granulocyte-macrophage colony-stimulating factor); as well as familial, overlap (with EGPA, in particular), idiopathic, and associated HES [91]. A therapeutically important subgroup within the HES variants are patients with an aberrant gene produced by fusion of the Fip1-like 1 (FIP1L1) gene with the platelet-derived growth factor receptor α (PDGFRA) gene [92]. Due to their specific aetiology, these patients respond to treatment with tyrosine kinase inhibitors such as imatinib [92].

Eosinophilic immune dysfunction is an important concept in HES. In patients without the FIP1L1/PDGFRA fusion gene, can we target eosinophils with IL-5/IL-5R therapies and not only discover an effective therapy, but potentially find some proof of concept that eosinophilic immune dysfunction is an important disease mechanism? Clinical trials of mepolizumab and benralizumab in HES have yielded positive results. A phase 3 study randomised patients with *FIP1L1-PDGFRA*-negative HES to treatment with mepolizumab 300 mg or placebo Q4W for 32 weeks added to existing HES therapy [93]. The proportion of patients who experienced a disease flare or who withdrew from the study was 50% lower in the mepolizumab group (28%) compared with the placebo group (56%; p=0.002). At the time of the primary assessment, mepolizumab had reduced mean eosinophil count by 92% from baseline compared with placebo. Earlier clinical data had demonstrated the steroid-sparing effects of mepolizumab in patients with HES [94]. In a phase 2 study that enrolled 20 patients with *PDGFRA*-negative HES and an absolute eosinophil count of ≥1000 cells·mm^−3^, benralizumab was effective in reducing eosinophil counts (the primary endpoint) [95]. The investigators also found that patients receiving benralizumab experienced reductions in skin lesions and decreases in tissue eosinophilia.

The efficacy of IL-5/IL-5R-targeted therapies in different HES variants remains to be fully characterised. Data from a long-term study of mepolizumab administered under compassionate use authorisation for severe, treatment-refractory HES give some indication of differences in treatment response among HES variants, with a greater likelihood of response observed in OCS-responsive patients who had idiopathic or overlap forms of HES [96]. In patients with lymphocytic variant HES, mepolizumab has been shown to have steroid-sparing effects; however, the efficacy of treatment in reducing eosinophil counts is suboptimal compared to patients with idiopathic HES [97].

As with EGPA, testing IL-5/IL-5R therapies in HES has helped in two ways: first, by proving that reducing eosinophil counts can be an effective strategy for this population of patients in desperate need of new therapies, and second, by providing proof of concept that eosinophilic immune dysfunction is indeed an important part of HES.

### Other systemic eosinophilic diseases

Eosinophilic gastrointestinal disorders encompass eosinophilic diseases of various components of the gastrointestinal tract including the oesophagus, as observed in EoE, the most prominent and well-studied condition in this group (table 2) [46]. EoE shares several commonalities with asthma, including aberrant eosinophil activation and recruitment, the prominence of IL-13 and epithelial dysfunction, and overlapping therapeutic targets such as the IL-4 receptor, IL-13, thymic stromal lymphopoietin, IL-5, and IL-5R [98, 99]. The common mechanistic underpinnings provide the proof of concept that eosinophilic immune dysfunction has the potential to span multiple organs. Although studies of IL-5/IL-5R-targeted therapies have been disappointing, whether this is due to greater involvement of other cell types in disease pathogenesis (*e.g*., mast cells) or the challenges of measuring efficacy in EoE clinical trials remains to be determined [100].

Eosinophils are found in a variety of skin diseases, including atopic dermatitis, contact dermatitis, eosinophilic cellulitis (Wells syndrome), bullous pemphigoid, and organ-restricted HES (table 2) [45]. The potential for eosinophil-targeted therapies, including anti-IL-5/IL-5R therapies, is still an active area of investigation, with some mixed results. Clinical trials in this therapeutic area can give insights into the extent to which eosinophilic immune dysfunction is involved in particular skin diseases. Overall, eosinophilic diseases with manifestations outside of the airways are the next frontier in understanding the breadth and depth of eosinophilic immune dysfunction.

## Conclusion

The findings presented here outline the importance of eosinophils in human disease, ranging from organ-system-limited diseases such as asthma and CRSwNP to more broadly systemic diseases such as EGPA and HES. The advent of eosinophil knockout animal models and eosinophil-targeted therapies in humans has enhanced our understanding of the roles played by eosinophils in disease and allowed the investigation of their possible involvement in health maintenance. Eosinophilic immune dysfunction appears central to exacerbation pathogenesis and disease control in severe asthma and is clearly involved in the aetiology of several other eosinophil-related conditions. There remain patient subsets in whom eosinophil depletion is insufficient to fully resolve disease manifestations (*e.g*., asthma non-responders, EGPA variants, COPD, CRSwNP), highlighting disease heterogeneity. For these conditions, endotyping will help identify patients who are likely to derive the greatest benefit from eosinophil-depleting/targeting therapies. Future studies that engage cross-disciplinary collaboration are key to recognising and understanding the role of eosinophils in health and disease.
